# Remimazolam-Based Total Intravenous Anesthesia for Cardiac Surgery in an Infant With a Pathogenic RYR1 Variant

**DOI:** 10.14740/jmc5405

**Published:** 2026-10-02

**Authors:** Lily Lindemann, Peter Winch, Brittany L. Willer, Joseph D. Tobias

**Affiliations:** a Heritage College of Osteopathic Medicine - Ohio University, Athens, OH, USA; b Department of Anesthesiology & Pain Medicine, Nationwide Children’s Hospital and the Department of Anesthesiology & Pain Medicine, The Ohio State University College of Medicine, Columbus, OH, USA; c Department of Pediatrics, The Ohio State University College of Medicine, Columbus, OH, USA

**Keywords:** Ryanodine receptor 1, Malignant hyperthermia, Remimazolam, Total intravenous anesthesia, Congenital heart disease, Pediatric anesthesia

## Abstract

The ryanodine receptor 1 (*RYR1*) gene encodes a protein channel located on the terminal cisternae of the sarcoplasmic reticulum of skeletal muscle, thereby playing a key role in regulation of skeletal muscle contraction. *RYR1* gene mutations may result in inherited neuromuscular conditions with a wide range of clinical manifestations including profound hypotonia, proximal muscle weakness, facial weakness, respiratory involvement, and orthopedic disorders (scoliosis, hip dislocation, and arthrogryposis). Affected individuals may also be malignant hyperthermia susceptible. We present an infant with *RYR1* gene mutation who presented for anesthetic care during cardiac surgery for congenital heart disease. The clinical spectrum of *RYR1* gene mutation disorders is presented, previous reports of anesthetic care reviewed, and options for perioperative care discussed.

## Introduction

The ryanodine receptor 1 (*RYR1*) gene is located on the long arm of chromosome 19 at position 13.2 (19q13.2). It encodes the RYR1 receptor, a homo-tetrameric calcium channel, that is located on the terminal cisternae of the sarcoplasmic reticulum (SR) of skeletal muscle, cardiac muscle, smooth muscle cells, and the endoplasmic reticulum of B-lymphocytes and cerebellar Purkinje cells [1]. Within the skeletal muscle, the RYR1 receptor plays a critical role in calcium release, making it a key component in the regulation of cytoplasmic calcium homeostasis and skeletal muscle contraction. Under normal conditions, the RYR1 channel opens in response to the depolarization of T-tubules, resulting in the release of calcium from the SR into the cytoplasm [2–4]. Within the cytoplasm, calcium binds to troponin, leading to tropomyosin movement, which uncovers sites for the interaction of actin and myosin, resulting in muscle contraction.

Mutations in the *RYR1* gene may lead to inherited neuromuscular conditions known as RYR1-related diseases. There is a wide spectrum of clinical manifestations in affected patients, including profound hypotonia, proximal muscle weakness, facial weakness, respiratory involvement, and orthopedic complications such as hip dislocations [5, 6]. Of anesthetic relevance, affected individuals carry a significant risk for malignant hyperthermia (MH) susceptibility [7]. We present an infant with a *RYR1* gene mutation who presented for anesthetic care during cardiac surgery for congenital heart disease (CHD). The clinical spectrum of *RYR1* gene mutation disorders is presented, previous reports of anesthetic care are reviewed, and options for perioperative care are discussed with a focus on the potential for MH susceptibility and use of the novel benzodiazepine, remimazolam, as a part of a technique for total intravenous anesthesia (TIVA).

## Case Report

Review of this case and presentation in this format followed the guidelines of the Institutional Review Board of Nationwide Children’s Hospital. Written consent was obtained for the use of deidentified patient information for publication.

A 2-month-old male infant with a known RYR1 genetic mutation presented for pulmonary artery (PA) banding for the management of an atrioventricular septal defect (AVSD). The patient was born at 41 weeks gestational age via Cesarean section. At approximately 2 weeks of age, he developed repeated bouts of emesis, and at 3 weeks of age, he presented to his primary care physician with irritability, poor feeding, and a facial rash, initially attributed to a viral illness. Imaging at that time revealed possible cardiomegaly and increased pulmonary vascularity suggestive of CHD, prompting further workup. Echocardiography confirmed a complete atrioventricular canal defect. As part of the cardiac diagnostic workup, a genetic panel was performed, which incidentally identified a pathogenic RYR1 mutation, which was interpreted by the genomics laboratory as being pathogenic and having an association with MH (missense variant p.Gly2434Arg). A nasogastric (NG) tube was placed at 4 weeks of age to support feeding. Subsequently, the patient developed patchy pulmonary infiltrates with suspected pulmonary edema and respiratory distress. Given concerns of excessive pulmonary blood flow related to his CHD, he was scheduled for PA banding. Outpatient medications at the time of PA banding surgery included enalapril, furosemide, hydrochlorothiazide, and spironolactone.

On physical examination, the patient was resting comfortably. He was a 2-month-old male infant with a weight of 3.51 kg and a length of 54.5 cm. Vital signs included a temperature of 97.9 °F, heart rate of 138 beats per minute, respiratory rate of 60 breaths per minute, blood pressure of 62/49 mm Hg, and oxygen saturation of 95% on room air. Auscultation of the chest revealed clear and equal breath sounds bilaterally with no respiratory distress. Cardiac examination revealed a regular rate and rhythm with a 3/6 systolic murmur appreciated throughout the precordium. Capillary refill time was 3–4 s. There was no known family history of MH or adverse anesthetic reactions. The patient had no prior surgical or anesthetic history. Preoperative laboratory evaluation revealed hyponatremia with a sodium of 126 mEq/L, likely in the setting of his diuretic regimen, and borderline hyperkalemia with a potassium of 5.3 mEq/L. A complete blood count and coagulation studies were within normal limits.

Given concerns for the RYR1 variant, avoidance of MH triggering agents was planned with the use of TIVA. Per departmental policy for MH, the patient was scheduled as a first case of the day and the anesthesia machine was flushed with a high flow of oxygen for 1 h. The operating room (OR) MH cart was nearby with a full supply of dantrolene. The patient was an inpatient at the time of surgery, with peripheral intravenous access already established the day prior to surgery. He was held *nil per os* (NPO) for 6 h and transported to the OR where standard American Society of Anesthesiologists’ monitors were placed. Anesthesia was induced with ketamine (2 mg/kg) and fentanyl (2.5 µg/kg). Rocuronium (2 mg/kg) was administered to facilitate endotracheal intubation. Following the induction of anesthesia and tracheal intubation, an arterial catheter was placed in the left radial artery and a central venous catheter was placed in the left internal jugular vein. Anesthesia was maintained with continuous infusions of remimazolam (10 µg/kg/min), remifentanil (0.1–0.2 µg/kg/min), and dexmedetomidine (0.2–0.5 µg/kg/h). Additional intraoperative medications included fentanyl (total dose of 10 µg/kg), cefazolin for surgical site infection prophylaxis, and dexamethasone. Temperature and end-tidal carbon dioxide remained stable throughout the case, with no concerns for MH. Pulmonary artery banding and ligation of the patent ductus arteriosus were performed without use of cardiopulmonary bypass. Intermittent doses of phenylephrine were administered to maintain the mean arterial pressure. During closure of the sternotomy, the chest wound was infiltrated with bupivacaine (0.25% with 1:200,000 epinephrine). Residual neuromuscular blockade was reversed with sugammadex. Total operative time was 3 h and 24 min. At the conclusion of the procedure, the patient’s trachea was extubated when awake, and he was transferred to the cardiothoracic intensive care unit (CTICU). Approximately 30 min following arrival to the CTICU, the patient struggled to clear secretions and developed acute respiratory decompensation with oxygen desaturation, bradycardia, and hypotension. The event was treated by bag-valve-mask ventilation, epinephrine (1 µg/kg), and reintubation of the trachea. After treatment and resolution of the acute event, the patient’s trachea was extubated later that day to high-flow nasal cannula. The specific etiology of the postoperative respiratory event was thought to be multifactorial, related to the patient’s underlying hypotonia with poor airway control and the residual physiologic effects of the surgical procedure and anesthetic agents. The patient had a prolonged postoperative hospitalization due to poor feeding and failure to thrive. At the time of finishing this case report, the patient is currently 5 months old and has had intermittent, yet recurrent bouts bloody stools, pneumotosis intestinalis, poor weight gain, and feeding intolerance concerning for possible necrotizing enterocolitis that has resulted in multiple hospital admissions. These have been attributed to possible pulmonary over-circulation resulting in gut ischemia so he is scheduled for cardiac catheterization. His respiratory status has stabilized without supplemental oxygen although there are concerns of tracheomalacia with plans for a bronchoscopy and airway examination during the cardiac catheterization. Concerns related to hypotonia and other neurologic issues are stable.

## Discussion

RYR1-related diseases can follow autosomal dominant, autosomal recessive, or de novo inheritance patterns. They represent the most common cause of congenital myopathy with a prevalence of at least 1:90,000 [5]. *RYR1* gene mutations cause a spectrum of neuromuscular disorders with symptoms that include profound hypotonia, proximal muscle weakness, facial weakness, respiratory involvement, and orthopedic complications such as hip dislocations. In addition, there is a known association of RYR1 mutation with MH susceptibility [6].

MH is a pharmacogenetic disorder triggered by volatile anesthetic agents and succinylcholine, related to abnormalities in the RYR1 receptor and the transmembrane movement of calcium. The hypermetabolic crisis results from the rapid and uncontrolled release of calcium from the SR into the cytoplasm of skeletal muscle cells. Given the susceptibility of patients with specific RYR1 mutations to MH, avoidance of volatile agents and use of TIVA is recommended.

Remimazolam is a short-acting, ester-metabolized benzodiazepine that received approval by the United States Food & Drug Administration (FDA) in July 2020 for sedation of adult patients during invasive medical procedures lasting less than 30 min. Following its release for clinical, its perioperative applications have expanded to include administration as both a primary agent and as an adjunct to general anesthesia [8–10]. Although propofol remains the most commonly used anesthetic agent during TIVA, remimazolam may offer specific advantages, especially in patients with CHD, as clinical experience has suggested that it may result in a lower incidence of adverse hemodynamic effects than propofol [11]. Anecdotal experience has demonstrated its efficacy and hemodynamic stability in pediatric patients with CHD [12].

The potential safety of remimazolam in MH susceptible individuals has been suggested by both in vitro data and clinical experience (Table 1). Using an in vitro model, the responsiveness to caffeine was compared in HEK-293 cells expressing wild-type RYR1 with that of mutant RYR1 following perfusion with remimazolam or propofol [13]. Despite exposure to concentrations higher than those encountered clinically, neither remimazolam nor propofol influenced the caffeine-induced increase in intracellular calcium concentration. Subsequently, the intracellular calcium concentration was studied in myotubules derived from patients with known MH mutations to evaluate the safety of remimazolam in MH susceptible individuals [14]. Muscle biopsies were obtained from 10 patients, and myotubules were isolated and cultured for analysis. While supratherapeutic concentrations of remimazolam were associated with increased intracellular calcium, no significant elevation was observed at clinically relevant concentrations, suggesting that remimazolam does not trigger the calcium dysregulation associated with MH at doses used in clinical practice.

**Table 1:** Reports of Remimazolam Administration to Patients With Possible Malignant Hyperthermia Susceptibility

First author and reference	Patient demographics	Surgical procedure	Anesthetic agents administered
Uchiyama et al [15]	26-year-old, 64 kg male	Endoscopic sinus surgery and septoplasty	Induction: remimazolam infusion (12 mg/kg/h) with fentanyl, remifentanil, and rocuronium (doses not specified). Maintenance: remimazolam infusion (1.5 mg/kg/h) with rocuronium, fentanyl, and remifentanil (doses not specified).
Kondo et al [16]	72-year-old, 71 kg male	Endoscopic sinus surgery and nasal septoplasty	Induction: remimazolam (0.1 mg/kg), fentanyl (50 µg), and rocuronium (50 mg). Maintenance: remimazolam and remifentanil infusions (doses not specified), intermittent rocuronium (10 mg) guided by NM monitoring; flumazenil 0.2 mg for emergence.
Blansky et al [17]	6-month-old, 5.9 kg female infant	Repair of atrioventricular canal	Premedication: oral midazolam (0.5 mg/kg). Induction: remimazolam (10–15 µg/kg/min) and dexmedetomidine (0.5 µg/kg/min); endotracheal intubation facilitated with fentanyl (25 µg) and rocuronium (5 mg). Maintenance: remifentanil (0.3 µg/kg/min) and dexmedetomidine (0.5 µg/kg/min). Milrinone (0.25 µg/kg/min) during rewarming. Additional agents during CPB: propofol boluses (2–4 mg/kg), fentanyl boluses (total 35 µg), nitroprusside infusion (1 µg/kg/min). Reversal of NM blockade: sugammadex (8 mg/kg × 2 doses).
Petkus et al [18]	6-year-old, 24.3 kg girl	Dental rehabilitation procedure	Induction: propofol (4 mg/kg). No NMBA. Maintenance: remimazolam (5–7 µg/kg/min) with propofol (50 µg/kg/min). Analgesia with morphine (1 mg) and ketorolac (0.5 mg/kg).
Zhu et al [19]	1-year-old, 9 kg boy	Orchidopexy	Induction: remimazolam (0.2 mg/kg) and alfentanil (10 µg/kg). Maintenance: remimazolam (1–2 mg/kg/h) and alfentanil (10–20 µg/kg/h). Caudal block with 0.25% ropivacaine.
Murao et al [20]	80-year-old, 49 kg male	Laparoscopic inguinal hernia repair	Induction: remimazolam (12 mg/kg/h), fentanyl (50 µg), remifentanil (0.5 µg/kg/min), and rocuronium (40 mg). Maintenance: remimazolam infusion adjusted to the BIS, remifentanil infusion, and intermittent rocuronium (10 mg) guided by NM monitoring.

BIS: Bispectral Index; CPB: cardiopulmonary bypass; NM: neuromuscular; NMBA: neuromuscular blocking agent.

In addition to the laboratory data supporting the safety of remimazolam in MH, there have been anecdotal reports of the use of remimazolam in three adults and three pediatric-aged patients with MH susceptibility [15–20]. In these cases, remimazolam was used as part of the TIVA technique with adjunctive agents including propofol, dexmedetomidine, and opioids (fentanyl, remifentanil, alfentanil, morphine). Although limited and still anecdotal, these reports provide preliminary information regarding the clinical utility and potential safety of remimazolam in MH susceptible patients.

### Learning points

As genetic testing becomes more routine in the workup of patients with congenital conditions such as CHD, there may be increased identification of patients with RYR1 mutations. In addition to the primary anesthetic concern, the potential association of RYR1 mutations and MH susceptibility, patients with RYR1 mutations present with a variety of neuromuscular symptoms. The Clinical Pharmacogenetics Implementation Consortium (CPIC) guideline recommends that individuals with RYR1 variants be managed with non-triggering anesthetic agents and be treated as MH susceptible regardless of whether a formal MH diagnosis has been established [21]. In these patients, TIVA using non-triggering agents is indicated with avoidance of volatile anesthetic agents and succinylcholine. Our case adds to the limited literature describing the anesthetic management of infants with RYR1 mutations undergoing cardiac surgery with the novel use of remimazolam for TIVA.

Remimazolam represents an emerging option for TIVA in MH susceptible patients, supported by both in vitro data and the growing number of clinical case reports [12–20]. Remimazolam has a half-life of 5–10 min and can be reversed with flumazenil, offering potential pharmacologic advantages over propofol [10, 11]. Additionally, clinical evidence has reported a lower incidence of cardiovascular depression with remimazolam when compared to propofol in adult patients. While remimazolam is not FDA-approved for use in pediatric-aged patients, there is a growing body of literature reporting its use in infants and children in various clinical scenarios [22]. However, its use in MH susceptible patients remains anecdotal with a limited number of case reports. Additionally, clinical data with remimazolam dosing parameters in the pediatric population remain limited, specifically regarding induction and bolus dosing. Given the limited data on bolus dosing for the induction of anesthesia in neonates and infants, we chose to use ketamine and fentanyl in our patient with remimazolam reserved for maintenance anesthesia.

## Data Availability

Any inquiries regarding supporting data availability of this study should be directed to the corresponding author.
